# Abdominal Compartment Syndrome Due to Large Ovarian Cystadenoma: A Case Report

**DOI:** 10.7759/cureus.31389

**Published:** 2022-11-11

**Authors:** Ghadah J Khormi, Raghd S Ageeli, Rahaf J Othathi, Sadeem M Bingasem, Mohammed Al Ghadeeb

**Affiliations:** 1 General Practice, Jazan University, Jazan, SAU; 2 Radiology, King Fahad Hospital, Al-Hofuf, SAU

**Keywords:** intra-abdominal hypertension, case report, abdominal pain, decompressive laparotomy, abdominal compartment syndrome, acute abdomen

## Abstract

Abdominal compartment syndrome is a rare emergency condition characterized by the development of organ dysfunction due to increased intra-abdominal pressure. Gynecologic conditions are an uncommon etiology of abdominal compartment syndrome. We report a case of a 35-year-old woman who presented with severe abdominal pain and vomiting. The patient had a history of long-standing gastroesophageal reflux disease. On physical examination, the abdomen was distended and tense, suggestive of acute abdomen. Computed tomography revealed a large abdominopelvic cystic lesion, arising from the ovary, causing a significant pressure effect on the abdominal viscera. The patient’s condition deteriorated and had an altered level of consciousness with hemodynamic instability. She was intubated and received inotropic support. Subsequently, a life-saving emergency surgical decompression was performed. The ovarian cyst was evacuated and yielded 10 liters of fluid. Histopathological examination confirmed the diagnosis of ovarian cystadenoma. The patient remained in the intensive care unit postoperatively and was discharged in a good condition after 14 days of hospitalization. The case emphasizes the importance of considering abdominal compartment syndrome in patients with a clinical picture of acute abdomen. Failure to recognize this condition can lead to multiorgan failure and death.

## Introduction

Abdominal compartment syndrome refers to a new onset of single or multiple organ dysfunctions due to increased intra-abdominal pressure [1]. It is classified into primary and secondary types. In the primary type, the increased intra-abdominal pressure is related to abdominopelvic etiology, such as abdominal trauma and acute pancreatitis. In the secondary type, the etiology of increased intra-abdominal pressure is not due to abdominopelvic pathologies, such as sepsis and high-degree burns [2]. For research purposes, abdominal compartment syndrome is often defined using an intra-abdominal pressure threshold of 20 mmHg [1]. However, for clinical practice, such a strict threshold of intra-abdominal pressure is not applied because abdominal compartment syndrome can develop at no specific intra-abdominal pressure [3]. Here, we report a case of abdominal compartment syndrome due to large ovarian cystadenoma in a young woman.

## Case presentation

We present the case of a 35-year-old woman who came to our emergency department with a complaint of severe abdominal pain and vomiting. The pain was sharp in nature and was rated 8 out of 10 in severity. It was diffuse but more severe in the lower part of the abdomen. She reported having this pain for the last two weeks, but it worsened progressively over the last two days. It was non-radiating, and she could not identify any aggravating or relieving factors. There was no change in bowel habits. The patient also complained of decreased appetite and generalized fatigue. The past medical history was remarkable for gastroesophageal reflux disease for which she was using daily omeprazole 40 mg. She did not undergo any previous surgeries. The family history was unremarkable.

On physical examination, the patient appeared drowsy and had no signs of respiratory distress. The vital signs revealed tachycardia (110 beats per minute), tachypnea (22 breaths per minute), and hypotension (98/60 mmHg) with normal temperature and oxygen saturation. Abdominal examination revealed a distended and tense abdomen with generalized guarding and absent bowel sounds. Laboratory investigations revealed a hemoglobin level of 12.5 g/dL, a leukocyte count of 10,500 cells/mm^3^, a normal platelet count, a creatinine level of 2.1 mg/dL, and normal liver enzymes.

**Figure 1:**
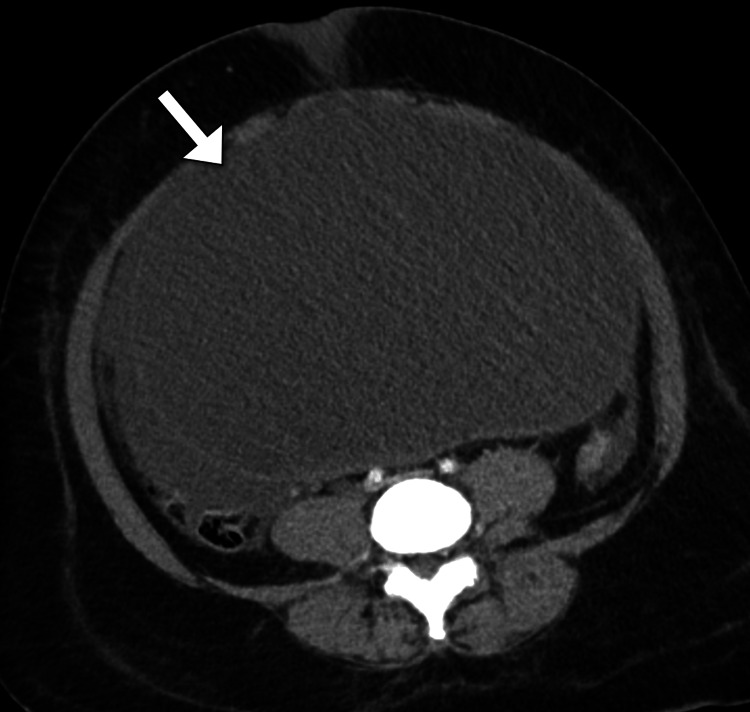
Axial abdominal CT image demonstrates a large unilocular cystic lesion (arrow) occupying most of the abdominal cavity. CT: computed tomography

**Figure 2:**
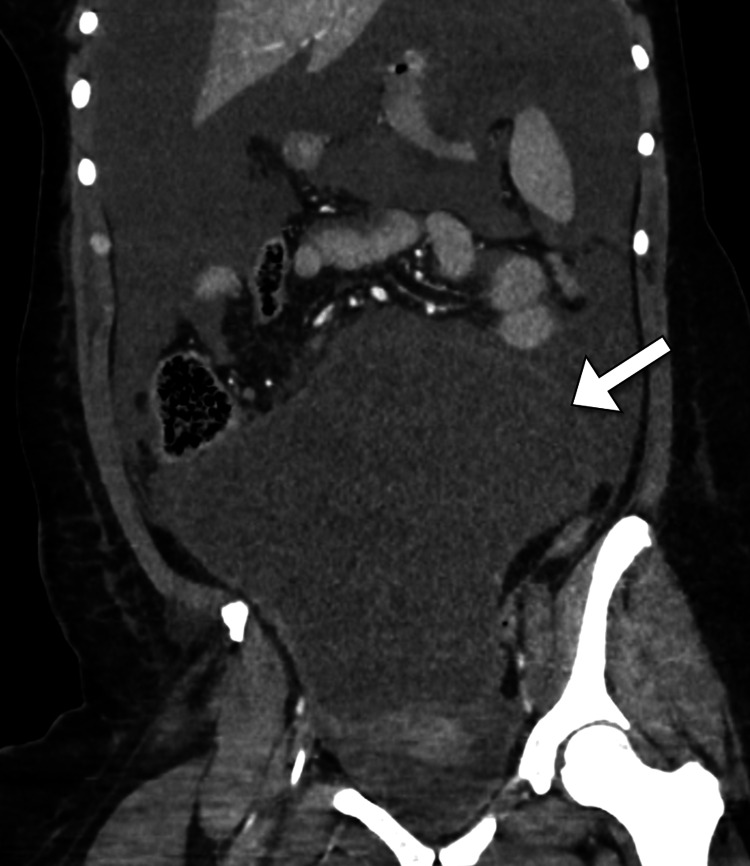
Coronal CT image of the abdominopelvic cavity shows a large cystic lesion (arrow) that is likely arising from the ovary with associated large intra-peritoneal free fluid. CT: computed tomography

Despite the resuscitation with intravenous fluid therapy, the patient developed a progressive worsening of her symptoms and had an altered level of consciousness. The patient was intubated with mechanical ventilation (maximum settings) and received inotropes to maintain blood pressure. The patient had decreased urine output. The clinical impression of the surgery team was suggestive of a perforated viscus. Subsequently, a computed tomography scan was performed to confirm the diagnosis and identify the possible site of perforation (Figures 1-2). Unexpectedly, the scan demonstrated a large abdominopelvic cystic lesion and a massive amount of intraperitoneal free fluid in the abdomen and pelvis. The lesion measured 25 x 17 x 18 cm (anteroposterior x mediolateral x craniocaudal) in maximum dimensions and had no septations or solid components. The lesion was found to be inseparable from the right ovary. The lesion exerted a mass effect on the visceral organs and the inferior vena cava, which appeared narrowed. There was no evidence of pulmonary or solid organ metastases. The intestinal loops were normal in size and showed no signs of intestinal obstruction. The clinical diagnosis of abdominal compartment syndrome was suspected given the hemodynamic instability of the patient. The intra-abdominal pressure was measured using an intra-vesical catheter and yielded a reading of 30 mmHg. Hence, the decision was taken to proceed with a lifesaving emergency laparotomy. The high risk of death during the operation was discussed with the family.

**Figure 3:**
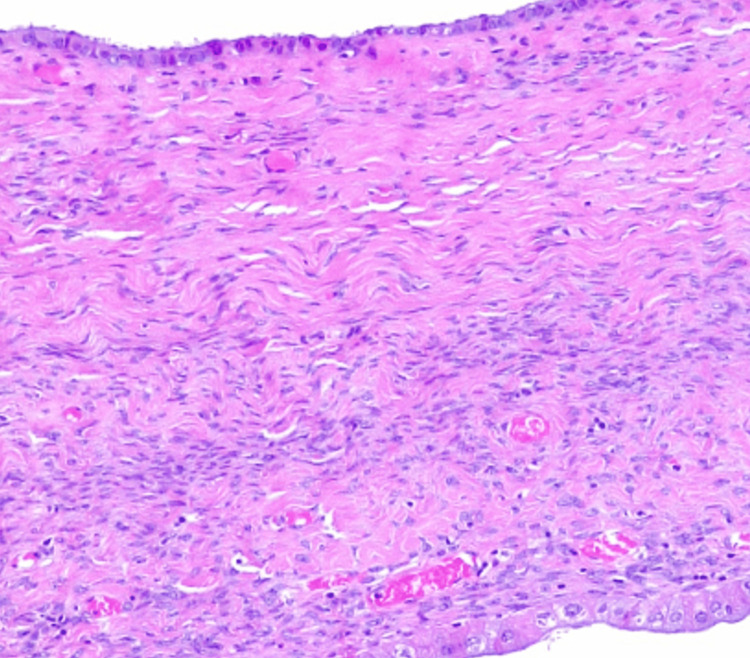
Histopathological image (hematoxylin and eosin) shows serous epithelial lining.

The patient underwent an emergency laparotomy under general anesthesia. During exploration, a markedly large cyst was noted arising from the ovary. A total of 10 liters were evacuated from the cyst. The fluid was dark in color suggestive of prior hemorrhage. Bilateral salpingo-oophorectomy was performed. The patient tolerated the operation. The incisions were closed. The patient did not need to have another abdominal surgery. Cytological examination of the fluid revealed no evidence of malignancy. Histopathological examination confirmed the diagnosis of ovarian serous cystadenoma (Figure 3). Postoperatively, the patient remained in the intestine care unit for seven days for supportive care. Her condition gradually improved after surgery. She was discharged after 14 days of hospitalization. At the one-month follow-up visit, the patient reported no active complaints. The patient was followed up for three months and underwent an abdominal ultrasound examination which showed no evidence of recurrence.

## Discussion

We present a rare case of abdominal compartment syndrome due to a massively large ovarian cyst, presenting with acute abdominal pain. The prompt decompressive laparotomy resulted in the survival of the patient who was in critical condition with hemodynamic instability.

Gynecologic conditions are considered an unusual etiology for the abdominal compartment syndrome with a limited number of reports in the literature [4,5]. For example, Sieloff et al. reported a lethal case of abdominal compartment syndrome due to rapidly enlarging ovarian carcinoma whose management was delayed because of the patient’s comorbidities and the atypical presentation with the absence of abdominal pain [5]. It should be noted that patients with abdominal compartment syndrome are typically very ill and are unable to convey their clinical symptoms. Additionally, the clinical signs from physical examination lack the accuracy to predict abdominal compartment syndrome [6]. In addition to large ovarian lesions, ovarian hyperstimulation syndrome is another gynecological etiology of abdominal compartment syndrome [4].

The definitive diagnosis of abdominal compartment syndrome requires measurement of intra-abdominal pressure. This pressure can be obtained indirectly by catheters in the stomach, bladder, or inferior vena cava. In the present case, we used an intravesical catheter, as per recommendations of guidelines [1]. Management of abdominal compartment syndrome includes supportive care and abdominal decompression. The purpose of supportive care is to reduce intra-abdominal pressure by evacuation of stomach content using a nasogastric tube, bladder decompression using a bladder catheter, and improving the compliance of the abdominal wall with analgesia and sedation. However, surgical decompression remains the definitive management [1,2].

Abdominal compartment syndrome is associated with significant morbidity and mortality. A prospective study involving 33 patients with confirmed abdominal compartment syndrome who underwent surgical decompression revealed a mortality rate of 36% at 28 days and 55% at one year. The study revealed that patients with advanced age and those who required mechanical ventilation were at higher risk of mortality [2].

The diagnosis of an ovarian cyst was readily made by computed tomography scan in the present case. This allowed for rapid life-saving emergency laparotomy. The differential diagnosis for intra-abdominal cystic lesions is limited. Such lesions include mesenteric, peritoneal, or gastrointestinal tract cysts [7]. These cysts can grow to a significantly large size as in the present case [7].

## Conclusions

We reported a case of giant ovarian cystadenoma resulting in abdominal compartment syndrome. The clinical symptoms and physical examination findings were inaccurate in predicting intra-abdominal compartment syndrome. The case highlights the importance of considering abdominal compartment syndrome in patients with a clinical picture of acute abdomen. Failure to recognize abdominal compartment syndrome can result in multisystem organ failure. Prompt surgical decompression is essential to reduce associated morbidity and mortality.
